# Management of donor site infections in split-thickness skin graft with water-filtered infrared-A (wIRA)

**DOI:** 10.3205/iprs000123

**Published:** 2018-06-12

**Authors:** Anas Aljasir, Thomas Pierson, Gerd Hoffmann, Henrik Menke

**Affiliations:** 1 Klinik für Plastische, Ästhetische und Handchirurgie – Zentrum für Schwerbrandverletzte, Sana Klinikum, Offenbach am Main, Germany; 2 Institut für Sportwissenschaften, Johann Wolfgang Goethe-Universität, Frankfurt am Main, Germany

**Keywords:** water-filtered infrared-A (wIRA), infection of donor site, skin transplantation

## Abstract

Infection of donor sites in split-thickness skin grafts is one of the complications of skin transplantation. Nutrition status and associated diseases play important roles in healing of donor sites. There are different ways used to treat infected donor sites. Water-filtered infrared-A (wIRA), as a special form of heat radiation with a high tissue penetration and a low thermal load to the skin surface, can improve the healing of acute and chronic wounds both by thermal and thermic as well as by non-thermal and non-thermic effects. Water-filtered infrared-A (wIRA) increases tissue temperature, tissue oxygen partial pressure and tissue perfusion. These three factors are decisive for a sufficient supply of tissue with energy and oxygen and consequently also for wound healing and infection defense. This was confirmed in a case with a late severe healing disturbance of the donor sites after skin transplantation.

## Zusammenfassung

Infektion der Spenderstellen bei Spalthauttransplantationen stellt eine der Komplikationen von Hauttransplantationen dar. Ernährungsstatus und Begleiterkrankungen spielen eine wichtige Rolle bei der Heilung von Spenderstellen. Es gibt verschiedene Wege, infizierte Spenderstellen zu behandeln. Wassergefiltertes Infrarot A (wIRA), eine spezielle Form der Wärmestrahlung mit hohem Eindringvermögen in das Gewebe bei geringer thermischer Oberflächenbelastung, kann die Heilung akuter und chronischer Wunden sowohl über thermische und temperaturabhängige als auch über nicht-thermische und temperaturunabhängige Effekte fördern. Wassergefiltertes Infrarot A (wIRA) steigert die Gewebetemperatur, den Gewebesauerstoffteildruck und die Gewebedurchblutung. Diese drei Faktoren sind entscheidend für eine ausreichende Versorgung des Gewebes mit Energie und Sauerstoff und entsprechend auch für Wundheilung und Infektiosabwehr. Dies wurde auch bei einem Fall einer schweren späten Heilungsstörung der Spenderstellen nach Hauttransplantation bestätigt.

## Introduction

Skin transplantation is the treatment for second and third degree burn wounds. Infection of donor sites is a possible complication of skin transplantation. We herein present a case of a 61-year-old male with infected donor sites that was managed with a daily non-adherent antimicrobial alginate dressing (Silvercell^®^) and irradiation with water-filtered infrared-A (wIRA). The aim of this report is to highlight the benefit of using water-filtered infrared-A (wIRA) in cases with infected donor sites.

## Case description

**Figure 1:**
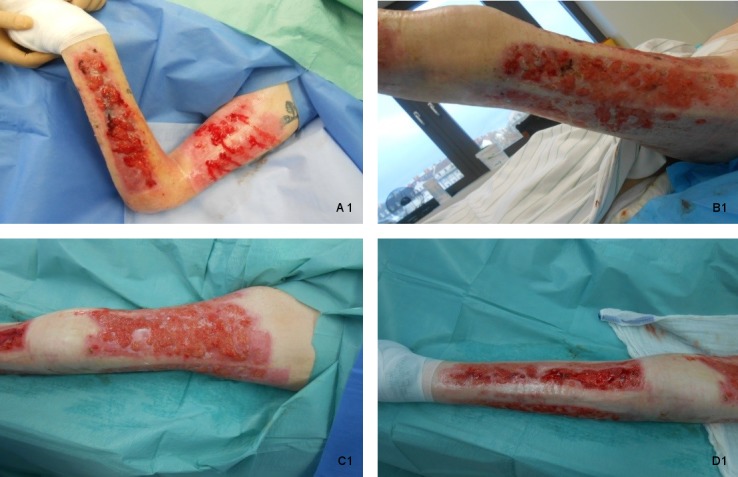
Severe healing disturbance with hypergranulation of the donor sites 8 months after split-skin transplantation operation in 61-year-old burn victim. (A 1: left upper arm and left forearm, B 1: lateral and posterior parts of left thigh, C 1: anterior part of left thigh, D 1: left lower leg)

**Figure 2:**
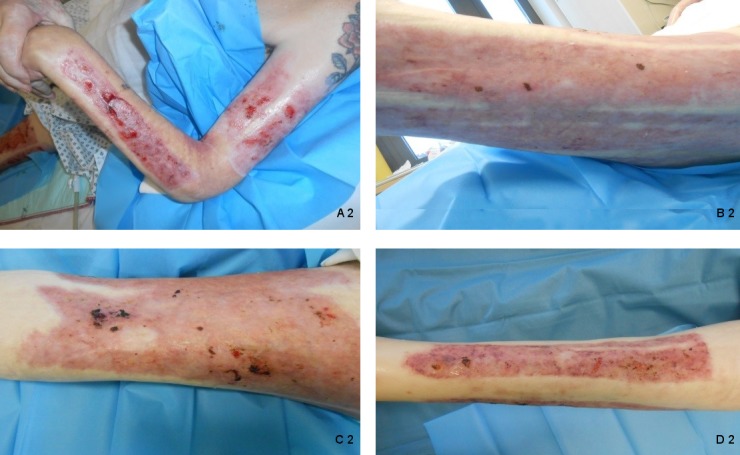
Donor sites after 5 weeks treatment with wIRA. (A 2: left upper arm and left forearm: nearly healed; B 2: lateral and posterior parts of left thigh, C 2: anterior part of left thigh and D 2: left lower leg: totally healed donor sites)

A 61-year-old male was referred to our burn center with burn injuries. The burn percentage was 20% Total Burn Surface Area (TBSA) and IIb-III degree, involving thorax, abdomen, and right arm. Patient was treated with split-thickness skin graft. We discharged the patient after successful management with healed graft and nearly healed donor wounds. 8 months later, the patient was referred to us from his primary care physician for management of infected open wounds in donor sites. Physical examination revealed infected area with hypergranulated tissue in donor sites, left upper limb and left lower limb (Figure 1 (Fig. 1)). Wound cultures showed *Staphylococcus* *aureus*, *Escherichia coli* and *Staphylococcus epidermidis* (Methicillin-resistent, MRSE) bacteria. Patient was a heavy smoker and suffered from multiple sclerosis. Up to our knowledge, neglect and inadequate wound care were responsible for the opening and secondary infection of donor sites. One day after admission we performed a surgical debridement and removed the hypertrophic granulation tissue. After the operation we started daily wound dressing with a non-adherent antimicrobial alginate dressing (Silvercell^®^) and irradiation with water-filtered infrared-A (wIRA). Water-filtered infrared-A (wIRA) protocols used at our department were radiations all wound areas three times daily with a 60 cm distance for 30 minutes with a “Hydrosun 505^®^” model (Hydrosun Medizintechnik, Müllheim, Germany). The patient tolerated this application well without any complaints. After 5 weeks the patient was discharged with healed donor sites left thigh and left lower leg and nearly totally healed sites left forearm and left upper arm (Figure 2 (Fig. 2)). 

## Discussion

Infected donor sites often complicate split-thickness skin graft (STSG). Many antimicrobials have been used in the management of infected donor sites, examples include wound contact dressings containing silver, gentamycin cream, fusidic acid cream, or silversulphadiazine cream. In this case we combined local antimicrobial treatment and irradiation with water-filtered infrared-A (wIRA) to improve the environment of the wound and wound healing. wIRA increases tissue temperature, tissue perfusion, and tissue oxygen partial pressure as basis of an increased metabolism and increased energy production. wIRA also has non-thermal and non-thermic effects (on cells and cellular structures and substances). In general wIRA decreases pain, inflammation, and hypersecretion and promotes infection defense and regeneration in a wide range of indications including acute and chronic wounds and infected wounds [1], [2], [3], [4], [5]. wIRA can be used i. a. for the promotion of healing of acute and chronic wounds (even an undisturbed “normal” wound healing can be improved: faster, with less pain and better cosmesis). 

Longer irradiation times per day result in larger effects. Thus, more frequent and longer lasting irradiations (e.g. several hours per day) with small irradiances are preferred to shorter lasting irradiations with higher irradiances [1], [2].

## Conclusions

Water-filtered infrared-A (wIRA) can optimize the environment of the wound and wound healing in split-skin donor sites. These will reflect in decreased healing time and an optimized treatment result and in a better cosmetic result. Therefore wIRA is recommended in addition to conventional treatment with antimicrobials. 

## Notes

### Competing interests

GH was working for the Dr. med. h.c. Erwin Braun Foundation, Basel, a charitable, non-profit Swiss scientific foundation approved by the Swiss Federal Administration. The foundation supports clinical investigation of waterfiltered infrared-A. The foundation was not involved in any content- or decision-related aspect of the case report. GH is not and was not employed by a commercial company and has not received fees or grants by a commercial company in the field of water-filtered infrared-A. Therefore, GH declares that no conflicts of interest exist according to the guidelines of the International Committee of Medical Journal Editors. The three other authors declare to have no competing interests.
